# The Use of Organoclays as Excipient for Metformin Delivery: Experimental and Computational Study

**DOI:** 10.3390/molecules29194612

**Published:** 2024-09-28

**Authors:** Sondes Omrani, Safa Gamoudi, César Viseras, Younes Moussaoui, C. Ignacio Sainz-Díaz

**Affiliations:** 1 Laboratory for the Application of Materials to the Environment, Water and Energy (LR21ES15), Faculty of Sciences of Gafsa, University of Gafsa, Gafsa 2112, Tunisia; sondes14omrani@gmail.com; 2 Faculty of Sciences of Gafsa, University of Gafsa, Gafsa 2112, Tunisia; younes.moussaoui@fsgf.ugaf.tn; 3 National Engineering School of Gafsa, University of Gafsa, Sidi Ahmed Zarroug, Gafsa 2112, Tunisia; safa.gammoudi@eniga.ugaf.tn; 4 Laboratory of Composite Materials and Clay Minerals, National Center for Research in Materials Science, TechnopoleBorjCedria, B.P. 73, Soliman 8027, Tunisia; 5 Department of Pharmacy and Pharmaceutical Technology, Faculty of Pharmacy, University of Granada, Campus de Cartuja s/n, 18071 Granada, Spain; cviseras@ugr.es; 6 Organic Chemistry Laboratory (LR17ES08), Faculty of Sciences of Sfax, University of Sfax, Sfax 3029, Tunisia; 7 Instituto Andaluz de Ciencias de la Tierra, Consejo Superior de Investigaciones Científicas (CSIC), Av. de las Palmeras 4, 18100 Armilla, Spain

**Keywords:** metformin, clay minerals, smectite, molecular modeling, drug release, adsorption

## Abstract

This work combines experimental and computational modeling studies for the preparation of a composite of metformin and an organoclay, examining the advantages of a Tunisian clay used for drug delivery applications. The clay mineral studied is a montmorillonite-like smectite (Sm-Na), and the organoclay derivative (HDTMA-Sm) was used as a drug carrier for metformin hydrochloride (MET). In order to assess the MET loading into the clays, these materials were characterized by means of cation exchange capacity assessment, specific surface area measurement, and with the techniques of X-ray diffraction (XRD), differential scanning calorimetry, X-ray fluorescence spectroscopy, and Fourier-transformed infrared spectroscopy. Computational molecular modeling studies showed the surface adsorption process, identifying the clay–drug interactions through hydrogen bonds, and assessing electrostatic interactions for the hybrid MET/Sm-Na and hydrophobic interactions and cation exchange for the hybrid MET/HDTMA-Sm. The results show that the clays (Sm-Na and HDTMA-Sm) are capable of adsorbing MET, reaching a maximum load of 12.42 and 21.97 %, respectively. The adsorption isotherms were fitted by the Freundlich model, indicating heterogeneous adsorption of the studied adsorbate–adsorbent system, and they followed pseudo-second-order kinetics. The calculations of ΔGº indicate the spontaneous and reversible nature of the adsorption. The calculation of ΔH° indicates physical adsorption for the purified clay (Sm-Na) and chemical adsorption for the modified clay (HDTMA-Sm). The release of intercalated MET was studied in media simulating gastric and intestinal fluids, revealing that the purified clay (Sm-Na) and the modified organoclay (HDTMA-Sm) can be used as carriers in controlled drug delivery in future clinical applications. The molecular modeling studies confirmed the experimental phenomena, showing that the main adsorption mechanism is the cation exchange process between proton and MET cations into the interlayer space.

## 1. Introduction

The application of clay minerals in the pharmaceutical field has increased in recent years, due to the growing success of recourse. The interaction of clays with drug molecules results in changes in the performance of the medicinal products once administered, allowing improvements in the stability and release of the loaded bioactives [1,2,3,4,5,6]. Clays and clay minerals are now considered interesting adsorbent materials, thanks to the size of the surfaces that they provide, the presence of electrical charges on these surfaces and, above all, the exchangeability of interlayer cations. The latter are the main elements responsible for hydration, swelling, plasticity and hydrophilic properties of these minerals. Indeed, clay minerals are deformable, transformable, adherent, flowing, slippery and fixing, and thus have many capacities among which are the transport, capture, and release of liquid and gaseous substances. In addition, their richness in trace elements promotes their use in health, well-being, cosmetics, and pharmaceuticals [7]. Different clay minerals have been proposed to modify drug delivery, including kaolinite and its polymorph halloysite [8]; fibrous clays such as palygorskite and sepiolite [9], as well as laminar clays [10]. Laminar clays correspond to the mineral group of smectites (Sm), including different clay minerals such as montmorillonite, hectorite and others. These minerals are phyllosilicates with a laminar TOT structure, in which each layer comprises an octahedral sheet (O) situated between two tetrahedral sheets (T). Between the layers, there are nanospaces capable of holding water and other organic or inorganic molecules. Sm is capable of adsorbing molecules, which can also occur through cation exchange in its interlayer space [11,12]. Its use as an excipient in the pharmaceutical field [13,14] and cosmetics is becoming interesting [15,16]. The Tunisian clay minerals have interesting properties for pharmaceutical and cosmetic applications [17,18]. Sm can be used as a vehicle to the gastrointestinal tract, and allows drugs to be adsorbed in its interlayer space, thus modifying its textural properties, such as its solubility, release, cation exchange capacity (CEC) and stability [19,20]. To date, several studies have been carried out to encapsulate drugs in Sm in order to modify the release profile of the pure drug [21].

**Figure 1:**
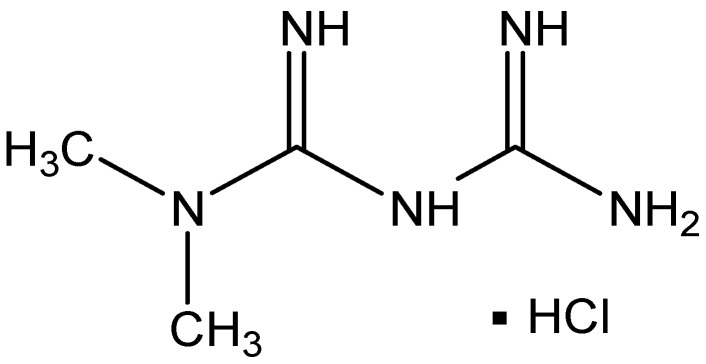
Structure of metformin hydrochloride.

Therefore, Sm is a good candidate for designing a new drug delivery system, such as metformine (MET), a hydrophilic drug used to treat type 2 diabetes (T2D) (Figure 1). MET, an antidiabetic known as 3 (diaminomethylidene)-1,1-dimethylguanidine, helps control blood sugar levels in people with diabetes mellitus [22]. MET belongs to the biguanide class and has been used for 30 years to treat hyperglycemia in type 2 diabetes mellitus (T2DM) [23]. Moreover, MET exerts its anti-diabetic effects primarily in the liver, through the inhibition of neoglucogenesis [24], and to a lesser extent in the intestine and muscles, leading to a reduction in hyperglycemia and circulating lipids, as well as an increase in insulin sensitivity. However, MET is a class III drug (biopharmaceutical classification system) with high solubility but low permeability, and subsequently, MET shows poor oral bioavailability. The design of drug delivery systems able to improve permeability is therefore required. In a previous work, MET interaction with two layered clays was studied [25]. The high loading capacity of a synthetic layered material (Laponite) was followed by a rapid release of the drug, requiring improvement of the delivery systems. The addition of a polymeric coating was achieved to optimize the release. This MET release systems included synthetic excipients and the coating of the loaded clay particles. To overcoming these limitations, the use of natural materials like clays and clay minerals would be desirable, as in other drugs [26]. Besides, we have used smectite from Tunisian mines in order to enhance the resilience of local markets.

Computational chemistry provides a useful approach to studying clay minerals [27,28] and the interactions of organics on solid surfaces at atomic scale [29,30]. Recently, this methodology has been applied to the adsorption of pharmaceutical drugs intercalated in clay minerals [25,26].

With these premises, one aim of this work is to assess a Tunisian clay in a purified and modified state as an excipient for drug delivery. Initially, we focused on the preparation and characterization of an organophilic clay prepared by the intercalation of a cationic surfactant HDTMA-Br. Secondly, we studied the adsorption of the active ingredient, metformin hydrochloride, by both purified and modified clays, as well as the release profile of this active ingredient. Computational molecular modeling calculations have confirmed the experimental behaviour, identifying the nature of intermolecular interactions and adsorption mechanism.

## 2. Results and Discussion

### 2.1. Characterization of Clays

**Figure 2:**
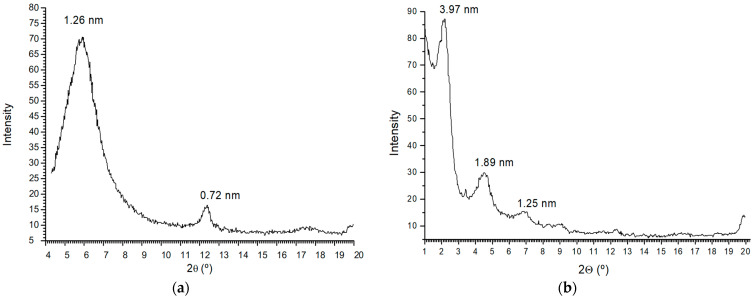
X-ray diffraction patterns of Sm-Na (**a**) and HDTMA-Sm (**b**).

Figure 2 shows the oriented aggregate powder XRD patterns of Sm-Na and HDTMA-Sm, prepared under optimized conditions. The characteristic peaks 2Ө of the (001) plane for Sm-Na and HDTMA-Sm are observed at 6.0° and 2.7°, with a basal spacing of 1.26 and 3.97 nm, respectively, for the most intense peaks. This peak shift to an increase in d-spacing indicates that HDTMA^+^ cationic surfactants were effectively intercalated into the interlayer space of Sm-clay by cation exchange, according to previous works [31,32,33].

In the XRD of the initial Sm-Na, a peak at 0.72 nm is observed, possibly due to a certain amount of kaolinite. In the HDTMA-Sm sample, several peaks with small intensity are observed at 1.89 and 1.25 nm, which can be assigned to a similar complex with another orientation or a partially exchanged organoclay and a certain amount of initial smectite that has not swelled, probably a small amount of illite. Therefore, a clear intercalation of surfactant is observed [34], but molecular modeling calculations will be necessary to determine the molecular orientation.

**Figure 3:**
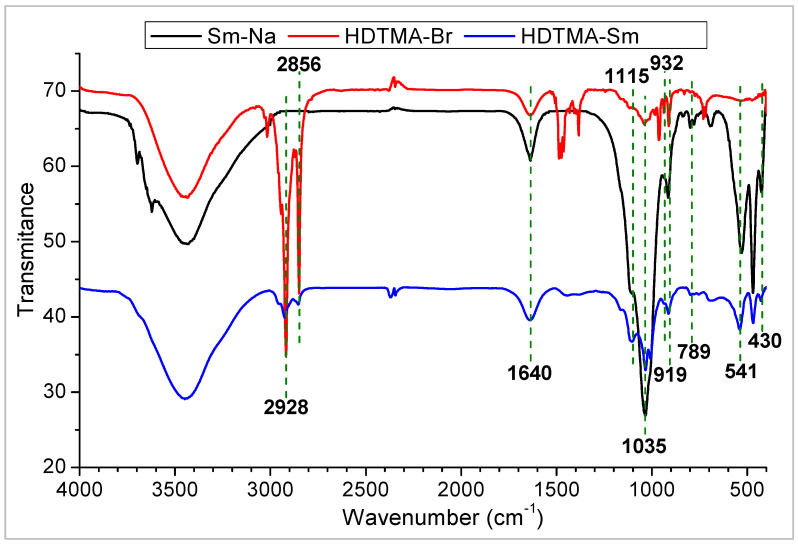
FTIR spectra of HDTMA-Sm (blue), HDTMA-Br (red), and Sm-Na (black).

The FT-IR spectrum (Figure 3) of Sm-Na presents bands located between 3700 and 3100 cm^−1^ that are assigned to the ν(OH) stretching vibration modes of the octahedral OH groups of smectite and the OH groups of the adsorbed water. The band superimposed at 1640 cm^−1^ is due to the δ(OH) bending vibration of adsorbed water. Two characteristic adsorption bands at 1115 and 1035 cm^−1^ are due to Si–O vibrations. The bands located at 932 and 541 cm^−1^ are due to the deformation vibration of (Al–Al–OH) and (Al–O–Si) in the octahedral layer and in the tetrahedral layer, respectively. The bands observed at 919 and 789 cm^−1^ are attributed to the δ(OH) vibration modes of the Al–OH and Mg–OH bonds [35]. These bands confirm the cation substitutions in the octahedral layer. The Si–O–Si group deformation vibration bands are located at 430 cm^−1^ [36]. By comparing the two spectra of Sm–Na and HDTMA–Sm clays, we notice the appearance of new peaks such as the bands of C–H alkyl bonds at 2928 and 2856 cm^−1^, which can only come from the structure of the surfactant agent found in the interlayer space of smectite. Hence, the FTIR spectroscopy results confirm the presence of alkyl ammonium ions (HDTMA^+^) in the modified clay.

**Figure 4:**
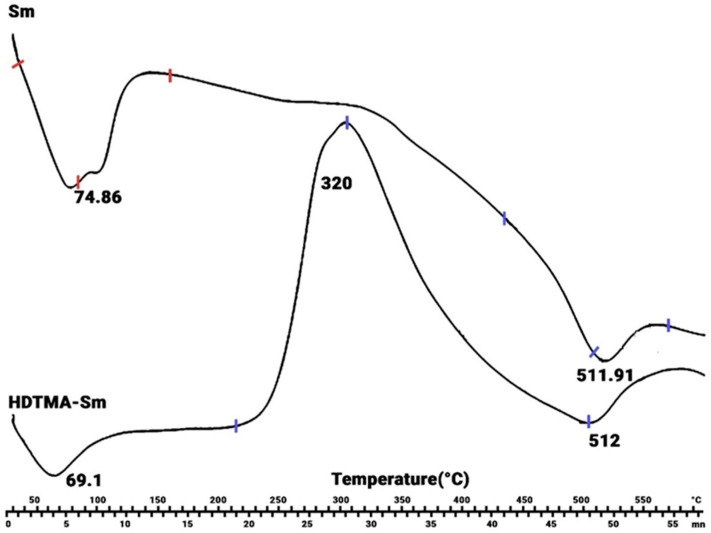
DSC profile of smectite before (up) and after (down) the formation of the HDTMA-Sm complex.

Comparing the DSC thermograms of Sm-Na and HDTMA-Sm (Figure 4), two endothermic peaks are observed at 50–120 °C and 450–580 °C, respectively. The first endotherm is due to the dehydration of the water on the clay surface, and the second one is due to the dihydroxylation of the structural OH groups of both materials. The DSC thermogram of HDTMA-Sm clay also indicates an exothermic peak at 250–400 °C corresponding to the decomposition of the organic matter (HDTMA^+^) present in HDTMA-Sm. This result confirms the intercalation of HDTMA^+^ cations in the clay interlayer space [36].

### 2.2. Characterization of Composite MET/Clays

**Figure 5:**
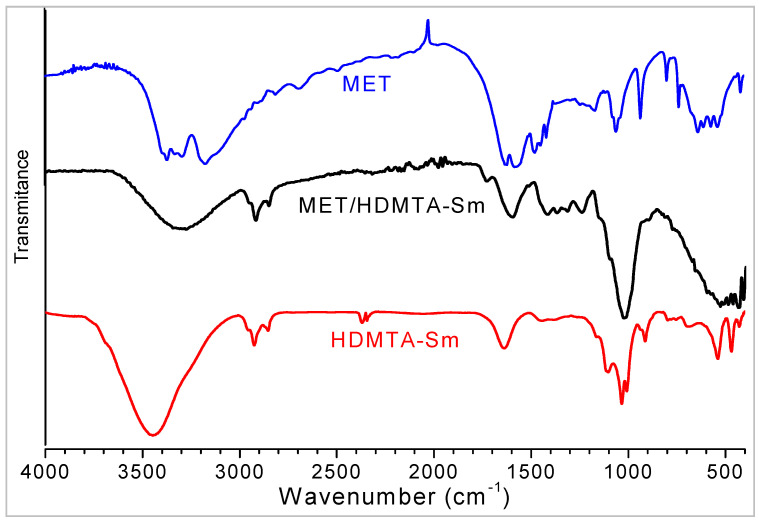
FTIR spectra of MET (blue), MET/HDTMA-Sm (black), and HDTMA-Sm (red).

The FT-IR spectrum of metformin hydrochloride (MET) shows the vibration modes of the N–H bonds of the C=N-H groups (Figure 5).

The bands observed at 3292 and 3369–3329 cm^−1^ are characteristic of asymmetric and symmetric stretching vibration ν(N–H), respectively. The bands at 2972 and 2812 cm^−1^ are attributed to the asymmetric and symmetric ν(C–H) vibrations of the methyl groups, respectively. On the other hand, MET exhibits an absorption band located at 1510 cm^−1^ due to the in-plane δ(NH_2_) bending vibration. The strong absorption of guanidines at 1580-1640 cm^−1^ is due to the ν(C=N) stretching vibration mode. Metformin is a biguanide and exhibits a strong absorption band at 1620 cm^−1^, attributed to ν(C=N). On the other hand, the vibrations of the C–N bonds of the aliphatic amine groups are generally weak and occur at 1058 and 1168 cm^−1^. In addition, the band at 570 cm^−1^ is due to C–N–C vibrations. The methyl groups exhibit two asymmetric δ(CH) bands at 1445 and 1480 cm^−1^ and symmetric δ(CH) bands at 1360-1420 cm^−1^. The band observed at 1477 cm^−1^ is assigned to the asymmetric δ(CH) vibration mode of CH_3_ groups [22].

By comparing the spectra of the purified clay before and after the intercalation of the drug (MET), no band shift was observed due to the small amount of absorbed drug with respect to the clay mineral. Nevertheless, the shift at lower frequencies of the broad band at 3600-3000 cm^−1^ in MET/HDTMA-Sm with respect to HDTMA-Sm indicates the presence of MET in the clay complex. Also, a shoulder at 1500 cm^−1^ indicates the presence of MET in the MET/HDTMA-Sm. In addition, a weak shift can be considered in the bands at 1480-1450 cm^−1^ of MET to 1400 cm^−1^ in the adsorption complex, indicating that there are some interactions between the N atoms of MET and the HDTMA-Sm support (Figure 5).

### 2.3. Effect of pH on MET Loading by Clays

**Figure 6:**
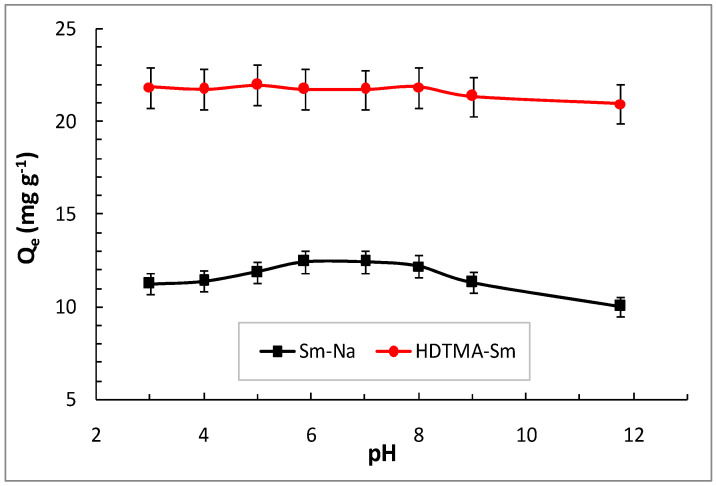
Effect of pH on the adsorption of MET in the smectite forms.

The loading of MET on the two clays was carried out at different pH values ranging from 3.0 to 12.0 using an initial MET concentration of 200 mg L^−1^ (Figure 6). It can be seen that for the HDTMA-Sm clay, the adsorbed amount of MET is greater (21.731 mg g^−1^) than in Sm-Na (12.428 mg g^−1^). The study of the influence of pH showed that the intercalation is independent of pH for the organophilic clay HDTMA-Sm. A different behavior is noticed for the retention of MET by the purified Sm-Na clay. Indeed, we observed a slight increase in adsorption of MET by Sm-Na for acidic pH (3 to 7) and a slight decrease in the quantities adsorbed for basic pH (8 to 12) (Figure 6). These results can be explained by the cationic form of MET interacting with the negatively charged surface of the clay mineral and a cation exchange mechanism.

The independence of MET adsorption with pH by HDTMA-Sm clay can be explained by two mechanisms—hydrophobic interactions and cation exchange. The MET molecules are trapped between the hydrocarbon chains of the surfactant in the interlayer space of the organophilic clay by the hydrophobic interactions. For the second mechanism, the MET molecules are cation-exchanged; however, we cannot know the mechanism of this process because the surfactants stabilize into Sm, and the cation exchange can probably occur with a proton cation of the interlayer water. Molecular modeling studies can give us additional information for a better understanding of this process.

### 2.4. Drug Binding Isotherm

**Figure 7:**
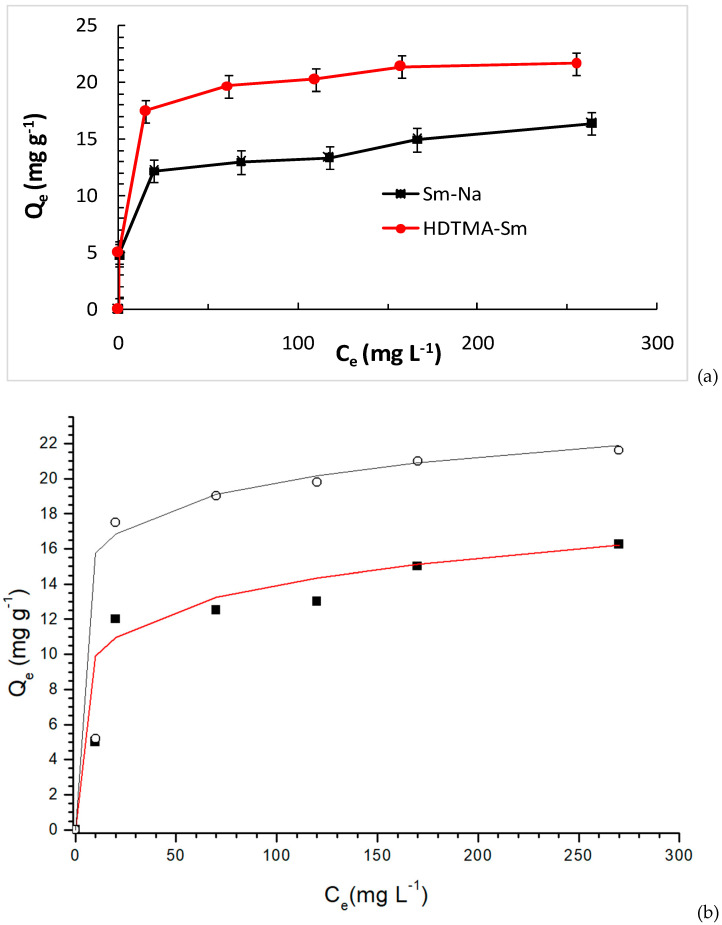
(**a**) MET adsorption isotherms on Sm-Na and HDTMA-Sm at 298 K. (**b**) Comparison of experimental data (Sm-Na in solid symbols, and HDTMA-Sm in hollow symbols) and the results of the Freundlich model (red line for Sm-Na, and black line for HDTMA-Sm).

Isothermal experiments were performed to investigate the effect of MET concentration on load capacity at a pH close to the optimum pH. The experimental binding isotherms of MET on Sm-Na and HDTMA-Sm at 298 K are shown in Figure 7. 

Initially, the adsorption capacity of Qe increases with the increase in the initial concentration of the adsorbate (MET), and the isotherms show an L-type appearance (according to the classification of Giles [37]). Indeed, the adsorption capacity of MET reaches a maximum at 16.25 mg g^−1^ Sm-Na. For the HDTMA-Sm clay, the adsorption capacity reached 21.61 mg g^−1^.

The Langmuir and Freundlich isotherm models were explored to interpret the equilibrium isotherm data. The Freundlich model assumes heterogeneous surface energies, whereas the energy in Langmuir’s equation varies with surface coverage. The equation of the Freundlich isotherm can be given as follows:

(1) $$Q_{e}=K_{F}\;C_{e}^{\frac{1}{n}}$$

where *Q_e_* id the quantity adsorbed at equilibrium (mg g^−1^); *C_e_* is the concentration at equilibrium (mg L^−1^); *K_F_* is the constant taking into account the adsorption capacity (L g^−1^); and *n* is the constant taking into account the intensity of adsorption.

The Langmuir adsorption isotherm is based on the assumption of monolayer adsorptions on a surface containing a finite number of adsorption sites. The Langmuir adsorption is given as follows:

(2) $$\frac{Q_{e}}{Q_{m}}=\frac{K_{L}C_{e}}{1+K_{L}C_{e}}$$

where *Q_e_* is the quantity adsorbed at equilibrium (mg g^−1^); *Q_m_* is the quantity adsorbed at saturation (capacity of a monolayer) (mg g^−1^); *C_e_* is the concentration at equilibrium (mg L^−1^); and *K_L_* is the adsorption equilibrium constant (L mg^−1^).

In view of the results illustrated in Table 1, the Langmuir model effectively describes the adsorption of MET by Sm-Na. The maximum monolayer adsorption capacity (qmax) is estimated as 16.779 mg.g^−1^, which is slightly higher than the experimental value, 13.25 mg.g^−1^. These results seem to prove that the adsorption of MET on the surface of Sm-Na clay is not chemical adsorption due to the absence of a single adsorption site. On the other hand, the Freundlich model fits more suitably with the adsorption of MET by both Sm-Na and HDTMA-Sm systems. Thus, MET molecules could be adsorbed in polylayers due to the presence of multiple adsorption sites [38]. In the fact, isothermal parameters indicate a different mechanism of MET adsorption by the Sm-Na and HDTMA-Sm clays.

### 2.5. Adsorption Kinetics

**Figure 8:**
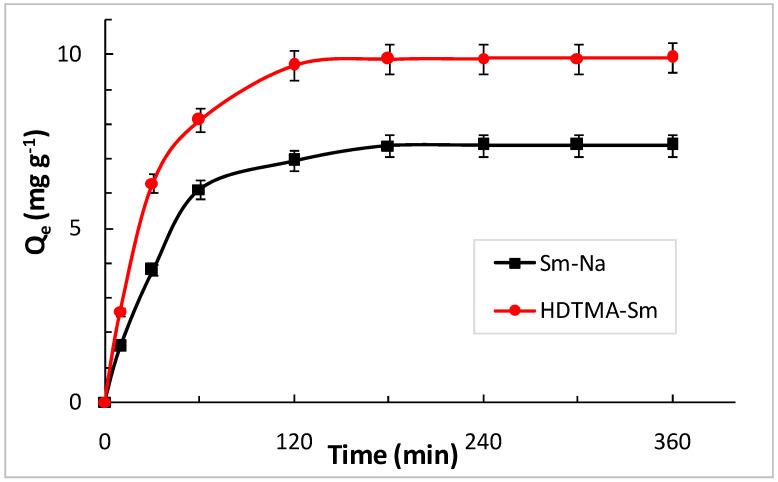
Kinetics of the MET absorption in Sm-Na and HDTMA-Sm at 298 K.

For the concentration of 100 mg L^−1^, the amount of MET adsorbed increases with the increase in contact time for both clays following different slopes (Figure 8). The evolution of the adsorption capacity of MET by Sm-Na and HDTMA-Sm shows the shape of the saturation curves. Adsorption maxima for MET are reached after two hours in these conditions, with amounts in the range of 7.384 and 9.884 mg g^−1^ for Sm-Na and HDTMA-Sm, respectively. This result is explained by the availability of and easy access to the adsorption sites, which facilitates the interaction between the MET molecules and the adsorbents.

The adsorption kinetics were evaluated using four models: pseudo-first-order, pseudo-second-order, intraparticle diffusion, and Elovich kinetic. The linear forms of the pseudo-first-order [39], pseudo-second-order [40], intraparticle diffusion [41,42], and Elovich [43] kinetic rate equations are given by Equations (3)–(6):

(3) $$\;Ln\left(Q_{e}-Q_{t}\right)=Ln\left(Q_{e}\right)-k_{1}t$$

(4) $$\frac{t}{Q_{t}}=\frac{1}{k_{2}Q_{e}^{2}}+\frac{t}{Q_{e}}$$

(5) $$Q_{t}=k_{3}t^{1/2}+C$$

(6) $$Q_{t}=\frac{1}{\beta }Ln\left(\alpha .\beta \right)+\frac{1}{\beta }Ln\left(t\right)$$

where *Q_t_* (mg/g) is the amount of dye adsorbed at time *t* (min), *Q_e_* (mg/g) is the equilibrium adsorption capacity, *k_1_* (1/min) is the rate constant of the pseudo-first-order equation, *k_2_* (g/(mg min)) is the constant rate of the pseudo-second-order equation, *k_3_* (mg/(g·min^1/2^)) and *C* (mg/g) are the rate constant and intercept of intraparticle diffusion equation, and *α* (mg/g min) and *β* (g/mg) are the initial adsorption rate and parameter related to the extent of surface coverage and activation energy of the Elovich equation.

The values of the adsorbed quantities Q_e_ calculated by the pseudo-second-order model are close to the experimental quantities (Table 2). Moreover, the values of R^2^ for the second model are significant and greater than 0.99. These results prove that the process of the adsorption of the MET by both clays, Sm-Na and HDTMA-Sm, follows the pseudo-second-order model.

### 2.6. Influence of Temperature

**Figure 9:**
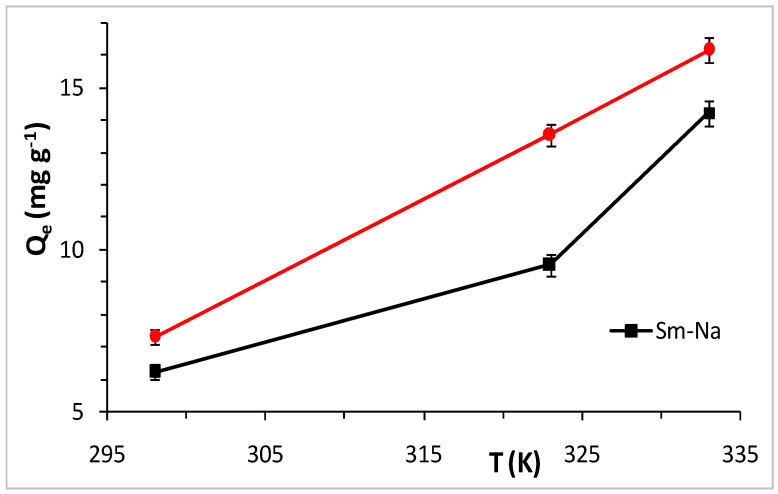
Influence of temperature on the adsorption of MET by clays. Black line for Sm-Na and red line for HDTMA_Sm.

The adsorption of MET increases with the increase in temperature, with maximum quantities of 6.233 and 7.301 mg g^−1^ for Sm-Na and HDTMA-Sm, respectively, at 298 K (Figure 9) being consistent with previous works [44].

Thermodynamic parameters, Gibbs free energy (ΔG_ads_), enthalpy (ΔH_ads_) and entropy (ΔS_ads_) were calculated, finding negative values (Table 3), indicating that the reaction is spontaneous and exothermic. The low value of the standard enthalpy (−20.89 kJ mol^−1^) of Sm-Na indicates physisorption. On the other hand, the high value of the standard enthalpy (−89.39 kJ mol^−1^) of HDTMA-Sm leads us to propose another adsorption mechanism, namely, cation exchange between the cationic species of MET and the cations of the interlayer space of clay minerals.

### 2.7. Release of MET/Clay

**Figure 10:**
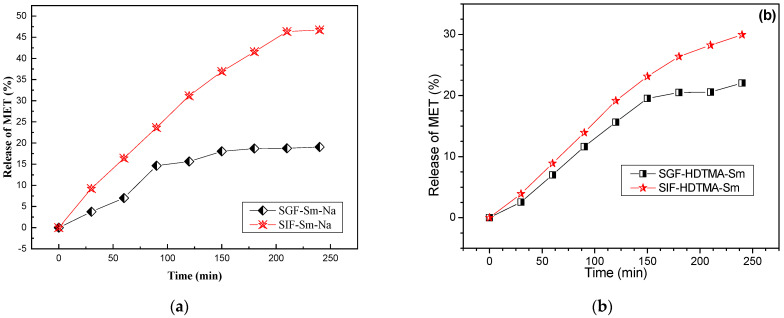
Release profiles in SGF (pH = 1.2) and SIF (pH = 7.4) of MET/Sm-Na (**a**) and MET/HDTMA-Sm (**b**).

The study of MET release by the hybrids MET/Sm-Na and MET/HDTMA-Sm was carried out in two solutions at different pH, SGF (simulated gastric fluid) at pH = 1.2 and SIF (simulated intestinal fluid) at pH = 7.4. The tests were carried out at 310 K (Figure 10).

For the purified Sm-Na clay, the release of MET depends on the pH of the reaction medium due to the high solubility of MET in the simulated intestinal fluid. After 220 min, the amounts of MET released from the composite (MET/Sm-Na) in the SGF and SIF solutions are 19.05% and 46.72%, respectively. The quantities of MET released at high pH are relatively large because the interactions between the molecules of MET and the Sm-Na clay are weak (physical interaction). This indicates that the release rate is significantly higher in SIF than in SGF. At low pH, MET is in a cationic form, and the interaction with the mineral surface is high. Hence, this clay system can be a good excipient for the controlled release of MET in oral administration according to previous works with clays and other drugs [14,45]. For the organophilic clay HDTMA-Sm, the maximal amounts of MET released from the composite (MET/HDTMA-Sm) after 220 min in SGF and SIF solutions are 22.05% and 29.94%, respectively. The results prove that the release is very low compared to that observed with the composite MET/Sm-Na, which indicates that the interaction of HDTMA-Sm with the drug is strong, and that the molecules of the active principle are trapped in the pores of the organophilic clay.

### 2.8. Computational Modeling

Some preliminary calculations were performed in order to validate our FF with different atomic charge distributions. The crystal structures of two polymorphs of HDTMA bromide [46] were calculated. We fully optimized both atomic positions and lattice cell parameters of both crystal structures of HDTMABr. In both crystals, the calculated crystal cell parameters are consistent with the experimental data (Table 4). Therefore, this result validates the use of this INTERFACE FF for the rest of our work. Besides this, we found that the polymorph CCDC1941222 is 11.90–12.57 kcal/mol more stable than the CCDC1941223 one.

The ammonium salt HDTMABr was optimally isolated within a periodical box (15 × 30 × 30 Å), being large enough for avoiding intermolecular interactions. Comparing this isolated structure with the crystal structure, we found that the packing energy of the CCDC1941222 crystal is −265.39 kcal/mol. On the other hand, this HDTMABr molecule was hydrated with water to explore the intercalation process in water, finding that the hydration energy was −109.41 kcal/mol.

**Figure 11:**
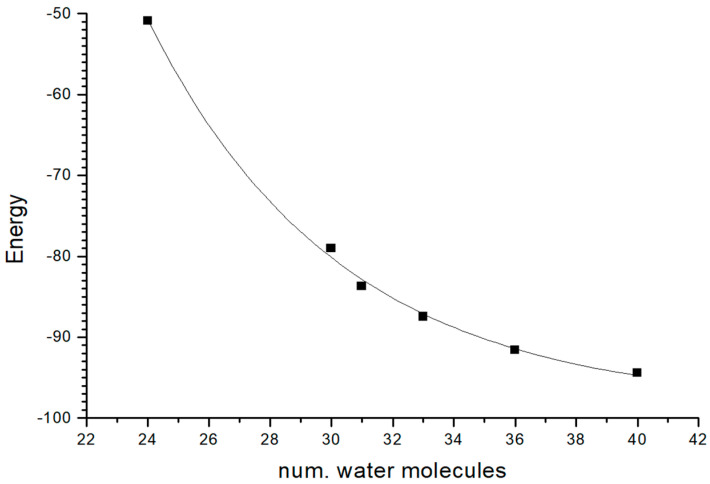
Relationship between the number of water molecules per 3x2x1 supercell, and the increase in hydration energy (kcal/mol) with respect to the model with 12 water molecules per supercell.

The montmorillonite model was fully optimized as a 3x2x1 supercell with 12 water molecules per supercell, and 2 water molecules per Na^+^ cation. The crystal cell parameters were consistent with the experimental data; however, the *d*(001) spacing was smaller than the experimental one. Hence, the amount of water was increased step by step, finding that the presence of 33 water molecules per 3x2x1 supercell reproduced the experimental value of d(001) spacing (Table 5). The adsorption of water molecules is energetically favorable, reaching a stabilization tendency with the amount of water molecules following an exponential curve (Figure 11).

**Figure 12:**
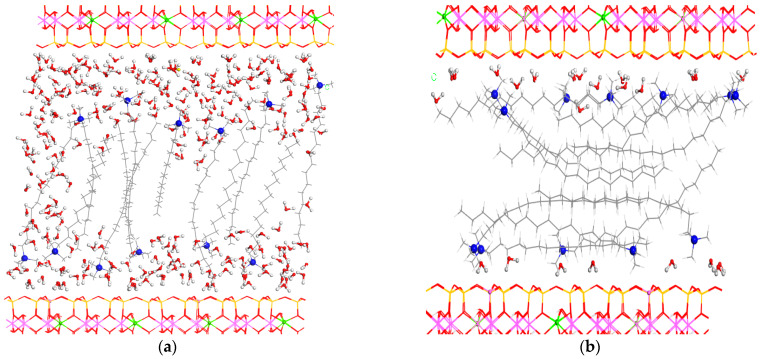
Optimized structures of the smectites intercalated with HDTMA in vertical (**a**) and horizontal (**b**) orientations with respect to the interlayer mineral surface. The H, O, N, C, Si, Al and Mg atoms are represented in white, red, blue, grey, yellow, pink, and green colors, respectively. This criterion is applied for the rest of the figures in this work.

For the adsorption of HDTMA^+^ cations, a cation exchange procedure was applied in a 3x4x1 supercell of smectite. In this supercell, there are 12 Na^+^ cations, which were exchanged by 12 HDTMA^+^ cations with the presence of two water molecules per cation. Two orientations were considered with all HDTMA chains, as follows: (i) a horizontal disposition, that is, parallel with respect to the internal smectite surface; or (ii) a vertical one, that is, parallel to the (100) plane. These chains were placed within an initial interlayer space of 40 Å in alternant orientations to each other in all cases. The full optimization of both models yielded lower interlayer spacings than the experimental one, being 27.0 and 33.0 Å for horizontal and vertical dispositions, respectively. The model with the surfactant in the vertical position was 109.59 kcal/mol more stable than that with HDTMA in a horizontal orientation. Although the total CEC of the smectite was completed with HDTMA, we had to add more water molecules to increase the interlayer space until reaching the experimental value observed above. Then, we added water molecules until reaching 100, 150, 200, and 300 water molecules per the 3x4x1 supercell of smectite. The full optimization of the smectite with 300 water molecules and 12 HDTMA^+^ cations per supercell yielded a *d*(001) spacing of 37.6 Å, being close to the experimental value. The water molecules were close to the mineral surfaces and the ammonium moieties (Figure 12).

**Figure 13:**
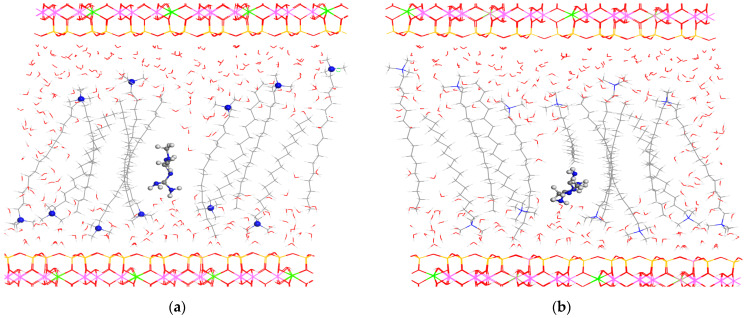
Optimized structures of MET intercalated in the HDTMA organoclay by cation exchange with a water proton (**a**) and by MET hydrochloride molecular adsorption (**b**). The atoms of the MET molecule are highlighted in balls.

Three processes of the absorption of MET can be considered, as follows: (i) a cation exchange where the metforminecation is exchanged by a HDTMA^+^ cation; (ii) the cation exchange between the MET and proton cations, and (iii) the adsorption of the metformin clorhydrate as a neutral adsorbate. The smectite with the HDMTA cations in a vertical disposition with 300 water molecules per supercell was chosen for the MET adsorption calculations. In all cases, the optimization of the intercalation complexes showed the MET molecule in the middle of the interlayer space among the surfactant chains with hydrophobic interactions (Figure 13). These three processes have been studied with our modeling calculations. The first two processes were exothermic, with −0.640 (i) and −188.08 (ii) kcal/mol, whereas the third process (iii) was not favorable (33.72 kcal/mol). Hence, the most probable adsorption process was the cation exchange mechanism between MET and a proton from the interlayer water molecule.

## 3. Materials and Methods

### 3.1. Chemicals

The montmorillonite sample used was a natural smectite from Maknassy (Sidi-Bouzid, Tunisia). The purification process was performed according to the classical method of Van Olphen [47], obtaining a homoionic Na smecite (Sm-Na). The purified clay Sm-Na was used with a CEC of 75.04 meq 100 g^−1^, and a high specific surface SBET = 92.74 m^2^ g^−1^. This clay was also modified via an organophilic treatment with the cationic surfactant hexadecyltrimethylammonium bromide (HDTMA-Br) (Sigma-Aldrich, St. Louis, Missouri, USA, analytical grade). Metformin hydrochloride (MET) (Sigma-Aldrich) was used as a pharmaceutical product with the molecular formula C_4_H_11_N_5_ • HCl.

### 3.2. Preparation of an Organophilic Clay

A batch method was used, which consists of bringing a solution of HDTMA bromide, with a concentration equivalent to 3CEC, into contact with a defined mass of Sm-Na clay. This procedure has yielded good results in previous works [17,18]. The suspension obtained was stirred for 24 h. Subsequently, the solid was filtered and washed several times with distilled water, filtered, and then dried at 333 K and ground.

### 3.3. Preparation of MET/Clay Composite

A sample of clay (0.1 g) in each case was added to MET solutions (10–300 mg L^−1^). The pH was adjusted by adding NaOH or HCl (0.1 M). During the study of the adsorption kinetics, the contact time varied from 10 to 360 min. The study of the influence of pH ranged from 3 to 12. The suspensions thus obtained were stirred for 2 h and then centrifuged. The final pH was also measured. The centrifuged solutions were analyzed by UV–visible spectrophotometry at λ = 233 nm, in order to determine the quantity of MET remaining at equilibrium. The adsorbed quantity (mg g^−1^) was determined by the difference between the initial concentration and that remaining in solution, by the following relationship:

(7) $$Q_{e}=\frac{(C_{0\;}-C_{eq})\times Vs}{m}$$

where *Q_e_* is the quantity adsorbed per unit mass of solid (mg g^−1^); *C*_0_ is the initial concentration (mg L^−1^); *C*_eq_ is the concentration at equilibrium (mg L^−1^); *Vs* is the volume of the solution (L); *m* is the mass of clay (g).

### 3.4. Release of MET/Clays

The in vitro study of MET release was carried out in two solutions: 

(a) pH 1.2 of the simulated gastric fluid (SGF)—50 mL of a 0.06 M HCl solution, containing 0.1 wt. % NaCl; (b) pH 7.4 of the simulated intestinal fluid (SIF) for 2 h—prepared by the addition of 0.15 g of NaOH, 0.2 g of NaH _2_PO_4_⋅H_2_O, 0.44 g of NaCl, 0.18 g of KCl and 0.4 g NH_4_HCO_3_ to 50 mL of ultrapure water. 

The clay samples under study (Sm-Na or HDTMA-Sm) were first saturated with a 0.1 g L^−1^ MET solution. The resulting suspensions were then stirred for 24 h. The MET-saturated clay was dried at 333 K. This clay was scored as either MET/Sm-Na or MET/HDTMA-Sm. Drug release was achieved by dispersing 0.1 g of MET-intercalated clay in 50 mL of SIF or SGF solution under continuous stirring at 400 rpm, at a temperature of 310 K. At 30 min intervals, 5 mL of release medium was withdrawn for concentration measurement, immediately followed by refilling with 5 mL of fresh dissolution medium, maintaining the total volume at 100 mL. The amount of MET released was analyzed using a UV–visible spectrometer at 233 nm.

### 3.5. Characterization Methods

Purified Sm-Na and modified HDTMA-Sm were characterized with reference to the XRD patterns of the sample, which were recorded within the 1–60° (2θ) range at a scanning speed of 2°/min by using a PANalytical X’Pert High Score Plus diffractometer with a wavelength of Cu radiation of 1.5406 Å. Fourier Transform Infrared (FTIR) spectra of clay and organoclay samples were obtained using the KBr pellet technique in a Perkin Elmer spectrophotometer (model 783) in the 4000–400 cm^−1^ range. Differential Scanning Calorimetry (DSC) was performed using a Diamond DSC Calorimeter (PerkinElmer). The sample was heated to 413 K at 10 K/min in N_2_ atmosphere. The Cation Exchange Capacity (CEC) of the clay mineral was determined using the copper ethylenediamine (EDA)_2_CuCl_2_ complex [48], and the Specific Surface Area Measurement (BET) was performed using the multipoint Brunauer–Emmet–Teller (BET) method at 393 K under a vacuum at 10^–3^ mm Hg for 3.5 h.

### 3.6. Computational Modeling

Two crystal structures of HDTMA bromide were taken from the crystallographic database (CCDC1941222 and CCDC1941223) [46]. The surfactant HDTMA bromide molecular structure was extracted from the crystal structure CCDC1941222. The MET molecular structure was taken from experimental crystallographic data [49], taking into account that this drug will be monoprotonated in aqueous solutions. For a more realistic work, several 3D periodical boxes with water and diverse salts participating in the intercalation process in the clay mineral were generated—HDTMA bromide, sodium bromide, chlorhydrate metformine, and sodium chloride. The Sm crystal model was developed using previously optimized structures [27]. We used a 3x2x1 supercell of Sm, where the chemical composition is Na_6_(Al_19_Mg_5_)(Si_47_Al)O_20_(OH)_4_. For the adsorption of HDMTA, we created a 3x4x1 supercell of Sm. Different amounts of water molecules were included in these models.

A force field (FF) based on empirical interatomic interactions applied recently in similar clay–organics systems, INTERFACE FF version 1.5, was used [50]. The geometry optimization calculations were performed using the Forcite code with periodical boundary conditions within the Materials Studio package [51], applying the Ewald summation method for the electrostatic calculation and van der Waals interactions in each periodical box with a cut-off at 15 Å. The generation of periodical boxes with water molecules was performed using Monte Carlo methods at 298 K with the same FF and the SPC water model. Several net atomic charges were used, derived from the Interface FF, Qeq (based on atomic electronegativities) and Gasteiger (based on orbital electronegativities) methods [51].

## 4. Conclusions

The adsorption of the pharmaceutical compound metformin hydrochloride, MET, on one Tunisian clay mineral with Na interlayer cations Sm-Na and onto an organo-clay, HDTMA-Sm, was studied under different experimental conditions, where the best results were estimated from the adsorption isotherm following 2 h of contact at room temperature (298 K) for an MET load of 21.61 and 13.25 mg per gram of HDTMA-Sm and Sm-Na, respectively. The results indicate the existence of clay–MET interactions. They also suggest the surface adsorption of MET onto Sm-Na and HDTMA-Sm. The most important interactions seem to be related to electrostatic forces and hydrogen bonds for Sm-Na and cation exchange and hydrophobic interactions for HDTMA-Sm. The equilibrium experimental data were modeled using the Freundlich isotherm, which indicate the heterogeneous adsorption characteristics of the MET/Sm-Na and MET/HDTMA-Sm system. The low and negative value of the Gibbs free energy (ΔGº), −10.75 and −19.46 kJ mol^−1^ for Sm-Na and HDTMA-Sm, respectively, suggest that the adsorption of MET is spontaneous and physical in nature. The computational molecular modeling calculations are consistent with these results, indicating that the HDTMA molecules are oriented perpendicularly to the interlayer space following the (100) direction, and the most probable desorption mechanism is by cation exchange between an MET cation and a proton cation from one interlayer water molecule. The in vitro release profiles of MET from the MET/Sm-Na and MET/HDTMA-Sm composites demonstrate that the clay releases the drug in a controlled manner. This behavior is superior in the modified clay, HDTMA-Sm, than in Sm-Na. The results show that this Tunisian clay can be a good candidate for the controlled release of MET. This may indicate its interesting applications in the pharmaceutical industry.

## Figures and Tables

**Table 1:** Isothermal (310 K) parameters for adsorption of MET on Sm-Na and HDTMA-Sm.

	Langmuir			Freundlich		
	Q_m_ (mg g^−1^)	K_L_	R^2^	n	K_F_	R^2^
MET/Sm-Na	16.779	0.00258	0.99	6.67	7.0	0.97
MET/HDTMA-Sm	21.739	0.00298	0.70	10.0	12.5	0.80

**Table 2:** Kinetics parameters for the adsorption.

Sample		Pseudo-First-Order			Pseudo-Second-Order			Intraparticle Diffusion			Elovich		
	Q_e_ exp (mg g^−1^)	k_1_ (mg/g min)	Q_e cal_ (mg g^−1^)	R^2^	k_2_ (mg/mg min)	Q_e cal_ (mg g^−1^)	R^2^	k_3_ (mg/mg min^1/2^)	Q_e cal_ (mg g^−1^)	R^2^	α (mg/g min^2^)	β (g/mg)	R^2^
Sm-Na	7.384	0.023	5.928	0.953	0.004	8.136	0.996	0.329	2.179	0.762	0.618	0.605	0.921
HDTMA-Sm	9.884	0.027	6.209	0.949	0.005	10.604	0.998	0.391	3.736	0.729	1.299	0.501	0.905

**Table 3:** Thermodynamic parameters of the adsorption of MET in the clay minerals studied.

Adsorbents	Temperature (K)	ΔG (kJ mol^−1^)	ΔH (kJ mol^−1^)	ΔS (kJ mol^−1^ K^−1^)	R^2^
**Sm-Na**	298	−10.758	−20.89	−0.034	0.950
	323	−9.908			
	333	−9.568			
**HDTMA-Sm**	298	−26.81	−89.39	−0.21	0.992
	323	−23.66			
	333	−19.46			

**Table 4:** Crystal cell parameters of HDTMA bromide optimized by using several atomic charges (distances are in Å and angles are in degrees).

Crystal Form	*a*	*b*	*c*	*α*	*β*	*γ*
**CCDC1941222**						
Exp	5.63	7.26	52.04	90.0	93.8	90.0
INTERFACE	5.53	7.01	52.25	90.0	95.3	90.0
Qeq	5.51	7.33	51.06	90.0	96.6	90.0
Gasteiger_q	5.56	6.97	52.22	90.0	94.7	90.0
**CCDC1941223**						
Exp	5.66	7.38	26.29	90.0	90.7	90.0
INTERFACE	5.32	7.54	25.35	90.0	94.4	90.0
Gasteiger_q	5.35	7.53	25.22	90.0	94.8	90.0

Exp: experimental crystal structure. INTERFACE: optimized with INTERFACE FF with charges assigned by this FF. Q_eq_: with INTERFACE and net atomic charges calculated with the Q_eq_ method. Gasteiger_q: with INTERFACE and atomic charges calculated with the Gasteiger method.

**Table 5:** Crystal cell parameters (distances are in Å and angles are in degrees) of the 3x2x1 supercell of montmorillonite with different contents of water compared with experimental data, and relative energy differences in kcal/mol.

Model	*a*	*b*	*c*	*α*	*β*	*γ*	*d*(001)	Δ*E ^a^*
Exp	15.71	18.17	12.78	90.7	99.7	89.8	12.6	
Sm12w	15.48	17.88	11.63	90.4	96.3	90.7	11.56	0.0
Sm24w	15.47	17.88	12.20	89.2	100.4	90.1	12.00	−50.90
Sm30w	15.48	17.89	12.39	85.3	97.6	90.1	12.28	−79.0
Sm31w	15.47	17.88	12.41	85.0	97.7	90.1	12.30	−83.71
Sm33w	15.47	17.88	12.82	83.1	101.1	90.1	12.58	−87.47
Sm36w	15.48	17.88	13.02	87.11	94.5	90.1	12.98	−91.60
Sm40w	15.48	17.88	13.86	82.9	98.7	90.1	13.70	−94.42

*^a^* Energy difference with respect to the model with 12 water molecules per supercell. Sm, Sm_Na; w, water.

## Data Availability

The original contributions presented in the study are included in the article, and further inquiries can be directed to the corresponding authors.
